# STATE HOME AND COMMUNITY-BASED SERVICES AND RESIDENTIAL CARE TRANSITIONS AMONG COMMUNITY-DWELLING OLDER ADULTS

**DOI:** 10.1093/geroni/igac059.2559

**Published:** 2022-12-20

**Authors:** Linda Chyr, Chanee Fabius, Emmanuel Drabo

**Affiliations:** Elevance Health, Baltimore, Maryland, United States; Johns Hopkins Bloomberg School of Public Health, Baltimore, Maryland, United States; Johns Hopkins Bloomberg School of Public Health, Baltimore, Maryland, United States

## Abstract

Medicaid home and community-based services (HCBS) provide integral health-related and personal care to support community-dwelling older adults. Growing literature suggests that states with more generous HCBS expenditures may delay or substitute costly nursing home care, but evidence is limited on the impact of HCBS spending on transitions to residential care settings like assisted living. This study determines the association of state Medicaid HCBS generosity—HCBS spending as a percent of total long-term services and supports expenditure—on the probability of incident transitions to residential care settings or nursing homes. Publicly available HCBS expenditure data was linked to a nationally representative sample of 7,197 community-dwelling older adults participating in the National Health and Aging Trends Study from 2011-2018. A discrete-time, competing risk regression model estimated the association between HCBS generosity and transitions from community to residential care settings or nursing homes, adjusting for sociodemographic, socioeconomic, and health factors. Most older adults remained in the community (93.7%). Incident transitions into residential care settings were twice as likely to occur compared to transitions to nursing homes (4.0% vs 2.3%, respectively). Older adults residing in states with higher HCBS generosity (one percentage point increase) are less likely to transition to nursing homes (relative risk ratio [RRR], 0.24; 95% Cl, 0.05-1.10; p< 0.1). Greater HCBS generosity was not associated with transitions to residential care setting. Assessing HCBS generosity on transitions elucidates important contextual factors affecting the movement of older adults across the care continuum.

